# Efficacy of Topical Hydroxypinacolone Retinoate‐Peptide Product Versus Fractional CO_2_ Laser in Facial Aging

**DOI:** 10.1111/jocd.16621

**Published:** 2024-10-12

**Authors:** Lucas Kruger, Kathryn Bambino, Kristine Schmalenberg, Uma Santhanam, David Orentreich, Catherine Orentreich, Jodi Logerfo, Claude Saliou

**Affiliations:** ^1^ Clinique New York New York USA; ^2^ Research and Development The Estée Lauder Companies Melville New York USA; ^3^ Orentreich Medical Group New York New York USA

**Keywords:** anti‐aging, cosmetic, hydroxypinacolone retinoate, laser, photodamage, retinoid, wrinkles

## Abstract

**Background:**

Many people are interested in addressing visible signs of aging with non‐invasive cosmetic treatments. Development of effective topical products will provide options to delay or support cosmetic procedures.

**Aims:**

This study assessed and compared the efficacy and tolerance of a topical product used over the course of 16 weeks to a single ablative laser treatment on women with moderate global photodamage on the face.

**Methods:**

Subjects in Cell 1 (Laser Cell) were treated over the entire face with a fractional CO_2_ laser system. Subjects in Cell 2 (Topical Serum Cell) were treated with a topical serum containing hydroxypinacolone retinoate and peptides over the entire face, twice per day for 16 weeks. The study was composed of 71 women, with 29 in the Laser Cell (mean age 56.2) and 42 in the Topical Serum Cell (mean age 55.0), between 40 and 65 years old. Expert grading was used to determine efficacy parameters.

**Results:**

Participants in the Topical Serum Cell achieved more significant improvement (*p* < 0.05) in Marionette lines, fine lines (global face), wrinkles (global face), wrinkles (crow's feet), nasolabial folds, texture, smoothness (tactile), global hyperpigmentation, lift, and photodamage compared to participants in the Laser Cell. Participants in the Topical Serum Cell achieved parity in the look of fine lines (crow's feet), forehead lines, glabella, firmness/bounce (tactile), skin tone evenness, radiance.

**Conclusions:**

While no statistically significant differences in tolerability were observed, treatment with the topical cosmetic product achieved parity or statistically better improvement in parameters compared to laser treatment at 16 weeks.

**Abbreviations**
CO_2_
: carbon dioxide
HPR: hydroxypinacolone retinoate
NS: no significant difference
PM: particulate matter
RARs: retinoic acid receptors
UV: ultraviolet

## 1 Introduction

The visible signs of skin aging are the result of deterioration and damage that accumulates in the layers of the skin: the epidermis, dermis, and subcutaneous tissue. While photodamage is considered one of the most impactful components in visual aging, the combination of ultraviolet (UV) light exposure, damage induced by environmental exposure to particulate matter (PM), along with the natural reductions in skin cell metabolism and renewal with age all contribute to visual aging [1]. With age, the impact of exposure to environmental aggressors on the skin's natural rejuvenation process increases, causing the skin to become thinner, drier, and less elastic [2]. Remodeling of and reduction in the amount of collagen in the dermis contributes to the overall changes in the structure of the skin as it ages. These structural and physiological changes manifest visibly as lines, wrinkles, and uneven pigmentation, among others.

Many people are interested in addressing visible signs of aging with non‐invasive cosmetic treatments. There is a continued increase in the demand for anti‐aging procedures and products [3, 4]. Those who do use professional services tend to use multiple procedures per dermatologist recommendation, which are typically more costly, require greater time commitment, and cause some discomfort compared to topical products.

Laser procedures, including ablative carbon dioxide (CO_2_) lasers, considered the gold standard of facial rejuvenation, remove a thin layer of tissue, and can be used as an alternative to dermabrasion and deep chemical peels for the resurfacing of photoaged skin. The most recent popular laser therapy is fractional CO_2_ laser resurfacing. Fractional ablative laser treatment generates microscopic columns of ablated tissue from the epidermis into the dermis in a defined pattern, with some devices having adjustable spot sizes and shapes allowing for treatment of more defined areas, maximizing improvements while minimizing downtime [5].

High efficacy cosmetic ingredients have been used for years in skincare formulations to minimize visible signs of aging. While investigations into topical formulations have shown improvement in visible signs of global photoaging, measured by both clinical grading and self‐assessment, when compared to a vehicle topical treatment [6], this study demonstrates the efficacy over time of a topical anti‐aging formulation containing signaling peptides and hydroxypinacolone retinoate compared to a single ablative laser treatment using clinical assessment parameters.

The topical serum is a water and silicone formulation that has been developed with a target to treat photodamage from three different angles: retinoids, peptides, and by helping restore hydration and moisture. Containing 9.5% peptides (acetyl hexapeptide‐8, palmitoyl hexapeptide‐12, palmitoyl tripeptide‐1, palmitoyl tetrapeptide‐7), 0.1% hydroxypinacolone retinoate, emollients, and hydrators, the formula has been designed for daily use with minimal adverse events while delivering skin anti‐aging benefits. Prior to clinical efficacy testing, a battery of safety and tolerance testing was performed to assess the skin tolerability and safety profile of the product. Safety and skin tolerance testing included human repeated insult test, phototoxity testing, sting panel, in vitro‐ocular, chamber scarification, in‐use dermatology testing as well as sensory and short‐term efficacy testing were performed. Panelists with different facial skin types (oily to dry) and with sensitive skin were included. Safety and tolerability were confirmed based on the results and observations of these tests, deeming this topical serum safe for use.

## 2 Materials and Methods

The study was conducted in Dallas, TX from September 2020 to April 2021. A minimum of 60 subjects meeting the eligibility criteria were required to be enrolled in the clinical trial, with at least 20 subjects in cell 1 (“Laser” Cell) and 40 subjects in cell 2 (Topical Serum Cell). Inclusion criteria included: being female between 35 and 65 years of age, Fitzpatrick skin type I–III; with moderate (score of 4–6.5 according to a modified Griffiths scale [7], where 0 = none and 9 = severe) scores for overall global photodamage on the entire face; having not had any facial treatments in the past 6 months and will withhold all facial treatments during the course of the study—including facials, facial peels, photo facials, laser treatments, dermabrasion, botulinum toxin, injectable filler treatments, intense pulsed light (IPL), acid treatments, tightening treatments, facial plastic surgery, or any other treatment administered by a physician or skin care professional designed to improve the appearance of firmness of facial skin; willing to provide written informed consent and able to read, speak, write, and understand English.

### 2.1 Informed Consent Form

The study was reviewed and approved by IntegReview Institutional Review Board (IRB) in Austin, TX, and an informed consent form (ICF), consistent with the requirements in 21 Code of Federal Regulations (CFR) 50.25 was given to each prospective subject before participation in any study procedures.

### 2.2 Subject Identification and Instructions

*Pre‐Study Instructions*

- Avoid application of any topical moisturizing products or other treatment products to the face for at least 2 days prior to baseline.

*“Laser” Cell*:

- Baseline to Day 10: Apply post‐care products. Use provided cleanser and moisture twice daily, morning, and evening. Use provided sunscreen during the day.
- Day 10 to Week 12: Discontinue use of the post‐care products. Use provided cleanser and moisture twice daily, morning, and evening. Use provided sunscreen during the day.

*Topical Serum Cell (Contains 9.5% Peptides (Acetyl Hexapeptide‐8, Palmitoyl Hexapeptide‐12, Palmitoyl Tripeptide‐1, Palmitoyl Tetrapeptide‐7) and 0.1% Hydroxypinacolone Retinoate)*:

- Apply two to three pumps of the test material twice daily, morning, and evening, on clean, dry skin. Use sunscreen during the day.

#### 2.2.1 Conduct of Study

Prior to the start of the study, prospective subjects were screened for eligibility requirements using an IRB‐approved script. Prospective subjects were informed of the pre‐study washout period and pre‐visit procedures. Prospective subjects were evaluated for study suitability and completed an informed consent form (ICF) and other paperwork, as described for baseline visit, prior to visiting the clinic. Prospective subjects were assigned specific times for visiting the clinical facility.

#### 2.2.2 Assessments

Prior to assessments, subjects rested in designated rooms within the clinical facility to acclimate to ambient temperature and humidity conditions for at least 20 min. Assessments were conducted at baseline, day 3 (cell 1 only), day 10, and weeks 4, 8, 12, and 16.

### 2.3 Laser Treatment

Subjects in Cell 1 were treated on the face with a fractional CO_2_ laser system using the specifications: Power: 5–10 W, Dwell: 200–400 μS, and Dot spacing: 1000 μm (5.3% coverage).

### 2.4 Clinical Grading of Efficacy Parameters and Imaging Procedures

The primary method of assessing the efficacy of the serum and the laser cell was performed using a modified Griffiths photonumeric 10‐point scale which illustrates and assigns numerical values to the severity of cutaneous photodamage starting from 0 (not visible sign of aging/photodamage) to 9 (severe signs of aging/photodamage) [7]. The scale is an objective assessment tool that incorporates photographs and numerical data commonly used in dermatology, particularly for evaluating the efficacy of treatments aimed at skin conditions including visible signs of aging such as lines, wrinkles, even skin tone, pigmentation, and texture. Dermatologist or expert graders use this scale through a systematic approach that combines both live in person assessment and/or photo documentation and numerical scoring to evaluate the skin conditions. They undergo rigorous training using scale manuals, hands‐on practice and training, assessment tests and calibration with other graders and continuous monitoring. In addition, they are usually blinded to the treatment and specifics of the study to ensure an objective evaluation and reproducibility. While many scales have been developed for dermatology, the modified Griffith is widely used in the cosmetic industry and offer practical assessments for evaluating visible signs of aging relevant for the consumer. In this study, 16 visible signs of aging parameters were assessed using this scale: marionette lines, fine lines Global face, fine lines crow's feet, wrinkles global face, wrinkles crow's feet, forehead lines, nasolabial folds, glabella, texture (visual), smoothness (tactile), firmness/bounce (tactile), skin tone evenness, global, hyperpigmentation, radiance, lift, and photodamage. These parameters were graded on the scale 0 to 9, with 0 = none (best possible condition), 1 to 3 = mild, 4 to 6 = moderate, 7 to 9 = severe (worst possible condition) with half‐point scores could be used as necessary to more accurately describe skin condition.

Subjects were enrolled having moderate score of 4–6.5 according to the scale and randomly placed into cell 1 or cell 2. Each cell's individual attribute mean score was comparable at baseline (Table 1).

**TABLE 1:** Comparison of parameters in laser and topical serum cells at baseline.

Parameter	Baseline		
	Cell 1: Laser	Cell 2: Topical serum	Mean difference at baseline
Marionette lines	4.47	4.49	0.02
Fine lines Global face	4.08	4.03	−0.05
Fine lines Crow's feet	2.79	2.99	0.2
Wrinkles Global face	4.91	4.92	0.01
Wrinkles Crow's feet	3.08	3.41	0.33
Forehead Lines	3.19	2.99	−0.2
Nasolabial folds	4.38	4.28	−0.1
Glabella	2.19	2.58	0.39
Texture	4.88	4.91	0.03
Smoothness (tactile)	4.39	4.56	0.17
Firmness/bounce (tactile)	4.72	4.9	0.18
Skin tone Eveness	4.78	4.72	−0.06
Hyperpigmentation	3.9	4.08	0.18
Radiance	5.06	4.95	−0.11
Lift	4.94	4.85	−0.09
Photodamage	5.25	5.13	−0.12

Clinical grading of efficacy parameters was performed by a blinded, expert, trained grader at baseline, day 10, and weeks 4, 8, 12, and 16. Photo documentation was performed at baseline, day 3 (cell 1 only), day 10, and weeks 4, 8, and 12. Subjects adopted neutral, nonsmiling expressions with their eyes gently closed, and were carefully positioned for each photograph. Digital images were taken of each subject's face (left, center, and right views) using VISIA CR photo station (Canfield Imaging Systems, Fairfield, NJ) with a Canon Mark II digital SLR camera (Canon Incorporated, Tokyo, Japan). Photos were used to grade parameters appearance of forehead lines, glabellar lines, fine lines in the crow's feet area, and wrinkles on the crow's feet area.

### 2.5 Tolerability Evaluations

Tolerability evaluations were performed at baseline, day 3 (Laser Cell only), day 10, and weeks 4, 8, 12, and 16. Local cutaneous tolerability was evaluated by assessing the signs of erythema, edema, dryness, and scaling and by subject reporting of burning, stinging, itching, tightness, and pain globally on each subject's face. Zero‐to‐three‐point scales were used for tolerability evaluations with half‐point scores used as necessary to better describe the clinical condition.

### 2.6 Statistics

The intent‐to‐treat (ITT) population was the primary population for all statistical analyses. Statistical analyses were performed on each cell separately compared to its baseline as well as Laser Cell versus Topical Serum Cell. For applicable parameters, comparisons between treatments/groups were made in terms of changes from baseline. The null hypothesis, that the mean change from baseline is equal between the two treatments/groups at post‐baseline time points, was tested using the Wilcoxon rank sum test as described in the statistical analysis plan table. All statistical tests were two‐sided at significance level alpha = 0.05 unless specified otherwise. No multiple corrections were considered in the study. Statistical analyses performed using SAS version 9.4 (SAS Statistical Institute).

## 3 Results

A total of, 71 women completed the study, 29 in the Laser Cell (mean age 56.2), and 42 in the Topical Serum Cell (mean age 54.7), between the ages of 42 and 65 years. Both Cells included Fitzpatrick skin types I–III. Participants in the Topical Serum Cell achieved more significant improvement (*p* < 0.05) in the appearance of Marionette lines, fine lines (global face), wrinkles (global face), wrinkles (crow's feet), nasolabial folds, texture, smoothness (tactile), global hyperpigmentation, lift, and photodamage compared to participants in the Laser Cell. Participants in the Topical Serum Cell achieved parity in the appearance of fine lines (crow's feet), forehead lines, glabella, firmness/bounce (tactile), skin tone evenness, radiance, (Table 2). Two additional cells including subjects with Fitzpatrick skin types IV to VI and with Asian subjects were also enrolled. Improvements obtained on all parameters were similar to those seen with the Topical Serum Cell except marionette lines, which were not significant on the Asian subjects (data not shown).

**TABLE 2:** Statistical analysis of comparison between laser and topical treatments after 16 weeks.

Parameter	Mean difference at 16 weeks	*p*	Significant difference in favor of
Marionette lines	0.20	0.014	Topical Serum
Fine lines Global face	0.26	0.014	Topical Serum
Fine lines Crow's feet	0.25	0.128	NS
Wrinkles Global face	0.26	0.007	Topical Serum
Wrinkles Crow's feet	0.22	0.043	Topical Serum
Forehead Lines	0.10	0.488	NS
Nasolabial folds	0.16	0.033	Topical Serum
Glabella	−0.02	0.811	NS
Texture	0.23	0.009	Topical Serum
Smoothness (tactile)	0.35	0.008	Topical Serum
Firmness/bounce (tactile)	0.15	0.058	NS
Skin tone evenness	0.11	0.317	NS
Global Hyperpigmentation	0.21	0.048	Topical Serum
Radiance	0.05	0.544	NS
Lift	0.24	0.005	Topical Serum
Photodamage	0.18	0.048	Topical Serum

Abbreviation: NS, no significant difference.

**FIGURE 1:**
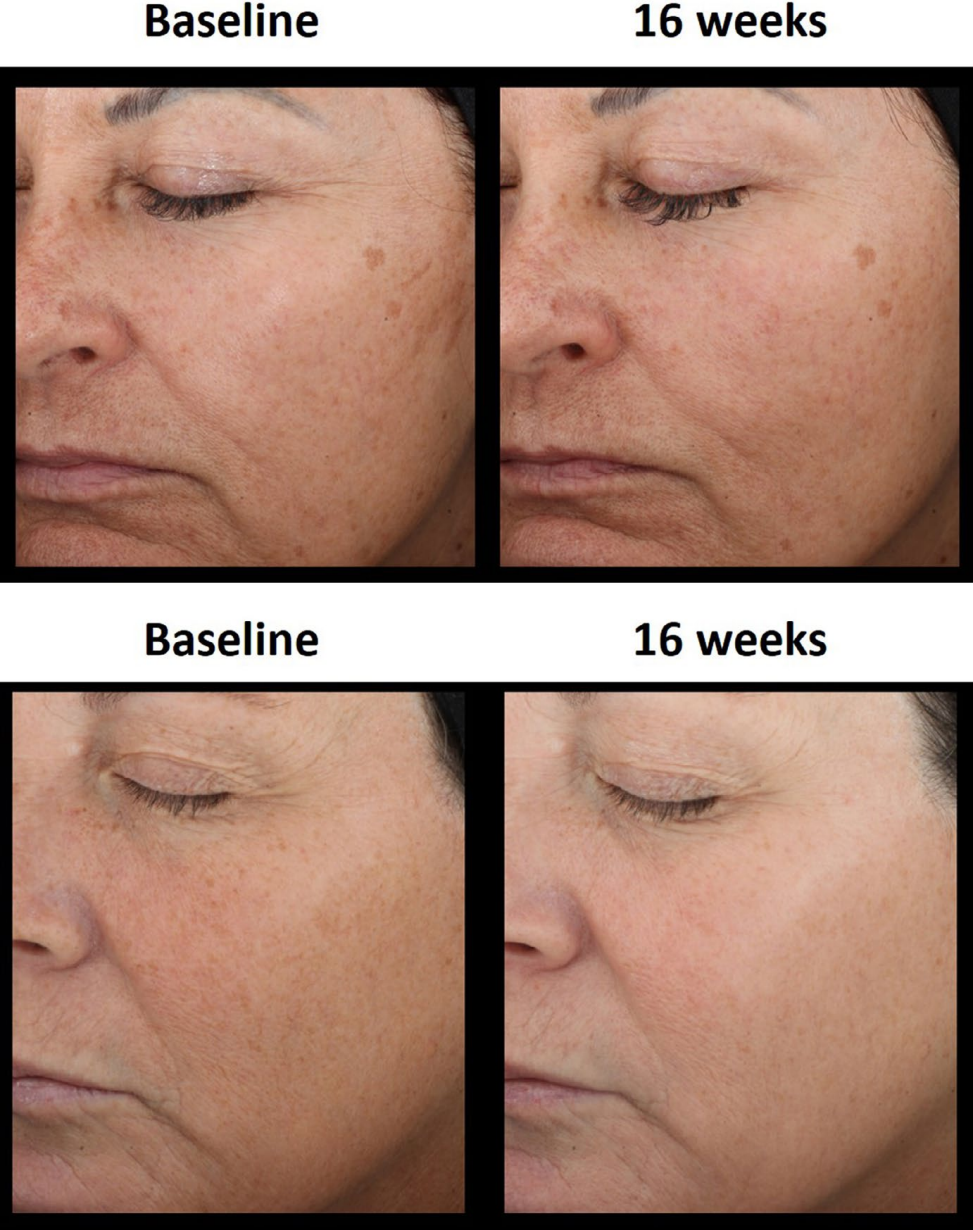
Before and after images of representative panelists in Laser Cell (panelist 41, top) and Topical Serum Cell (panelist 59, bottom).

**FIGURE 2:**
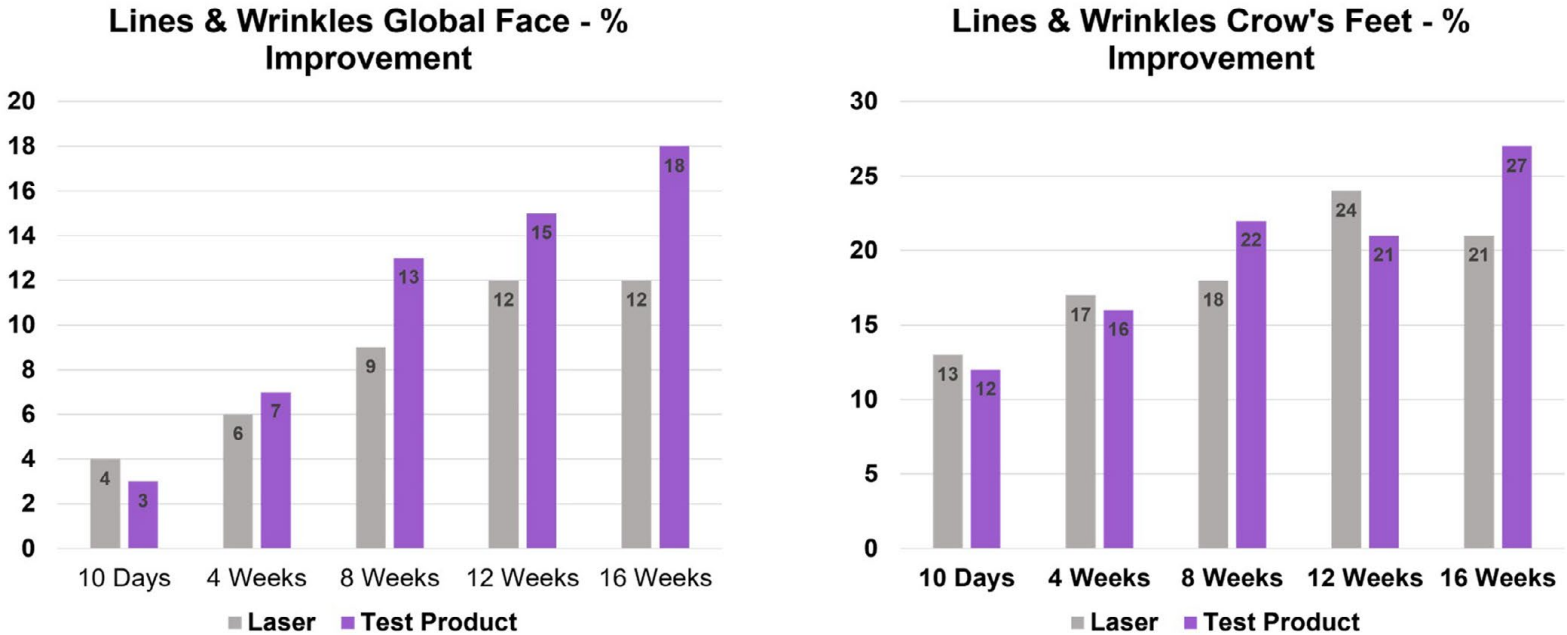
Progress of clinical improvement over time.

These results prove that the test product after 16 weeks of usage is as efficient as one Laser treatment in improving the appearance of marionette lines, fine lines global face, fine lines crow's feet, wrinkles global face, wrinkles crow's feet, forehead lines, nasolabial folds, glabella, texture, smoothness (tactile), firmness/bounce (tactile), skin tone evenness, global hyperpigmentation, radiance, lift, and photodamage (Figures 1 and 2).

## 4 Discussion

This trial compared the effects of a cosmetic topical treatment to a single laser treatment to improve the appearance of facial aging parameters in participants with mild to moderate photodamage. We compared a topical serum containing 9.5% peptides (acetyl hexapeptide‐8, palmitoyl hexapeptide‐12, palmitoyl tripeptide‐1, palmitoyl tetrapeptide‐7) and 0.1% hydroxypinacolone retinoate with a single treatment of a CO_2_ fractional laser after 16 weeks.

When selecting the comparative dermatologist treatment, it was imperative to understand which treatments would match the same theoretical benefits that the topical treatment was designed to affect. The gold standard in the industry since the mid 1990's for non‐surgical facial rejuvenation including the appearance of wrinkles, uneven skin tone, and global photoaging, has been the fractional CO_2_ laser. Fractional lasers have taken over the industry and have reduced recovery time while still delivering the same benefits of the classical ablative laser.

The use of fractional laser therapies for facial rejuvenation was first introduced in 2004. One previous long‐term observational study found that the effects of a single CO_2_ fractional laser treatment for facial photoaging could be maintained for up to 5 years [8]. Therefore, we are confident in this choice of an appropriate comparison for this study. Fractionated ablative CO_2_ lasers create microthermal zones, leaving uninjured columns of healthy tissue that aid in healing [9]. This process results in faster healing times and fewer adverse effects than traditional fully ablative CO_2_ laser therapy [9, 10, 11]. Duration of and time to peak results will depend on laser and protocols used. For this study, the peak result of the laser protocol used was in line with the timing included in the clinical evaluation.

Skin aging is a multifactorial process involving many different changes, so some people will require several different treatment modalities to achieve desired results. There are outstanding questions about the safety of combining different dermatological interventions, and many procedures carry the risk of significant downtime [12]. Patients with deeper skin tones are at high risk of dyspigmentation following treatment with dermatological procedures, including ablative lasers [13]. In contrast, topical cosmetics are generally considered with low risk of dyspigmentation. In this study, the efficacy of the Topical Serum tested was also separately assessed monadically on participants with Fitzpatrick skin types II–IV in Japan and Fitzpatrick skin types IV–VI in the United States with no dyspigmentation observed (data not shown); however, we did not directly compare the results in these groups to a cosmetic procedure.

In addition, there is evidence that a subset of patients seeking cosmetic procedures, including laser treatments, are poor healers and require more downtime than average following treatment with ablative lasers. Some explanations include that previous procedures such as peels or dermabrasion could make the skin more sensitive or that the resurfacing may go deeper than expected [13].

Due to common barriers to treatment experienced by patients with concerns over aging skin, the development and investigation of novel topical treatments addresses an unmet need. The market for anti‐aging topical products has grown dramatically in recent years as the availability of high‐performance alternatives and complementary approaches have evolved. Although the development of peptides for use in anti‐aging skin care began in the 1980's, innovation around new ingredients continues [14]. Many of these high‐performance ingredients have been adapted from the dermatology space including topical peptides, retinols, and exfoliants. Anti‐aging peptides in skincare can be categorized by their function and mechanism of action and can be powerful tool to improve the look of aging skin [14].

Retinol and retinoic acid have both been used for the treatment of skin aging [15]. Retinoids include natural and synthetic compounds that are derived from or synthesized to mimic the activity of vitamin A [2]. Retinoic acid receptors (RARs) are highly expressed in both keratinocytes and fibroblasts and signaling through these receptors plays a role in developmental and cellular processes [16]. Topical application of retinoids helps to enhance rejuvenation of the skin surface [15, 17]. As such, retinoids have been used in dermatology for decades to treat conditions such as acne and photoaging. More recently, they have moved into the cosmetic space [18, 19, 20]. Newer retinoid molecules offer increased stability for cosmetic formulations [18]. Hydroxypinacolone retinoate is part of the retinoic acid family but does not cause the high level of irritation typically associated with retinol and retinol derivatives [21, 22].

Advances in cosmetic formulation may allow people seeking dermatologic procedures to delay their first procedure and/or increase the time between procedures. They may also lead to the development of products that are more tolerable for consumers with darker skin tones and those with more sensitive skin.

The findings presented here demonstrate that treatment with a topical cosmetic product containing peptides and hydroxypinacolone retinoate is well tolerated and resulted in significant visual or tactile improvement in 16 parameters associated with photodamage and aging skin. Treatment with the topical serum provided comparable results versus a single treatment with a fractional CO_2_ laser system. The study protocol was designed to measure concerns that are addressed with fractional laser treatment. Typically, people see results with only one CO_2_ laser treatment, including 16 weeks post treatment; however, those with deeper wrinkles or significant scarring may need multiple treatments [23, 24, 25]. Within these confines, treatment with the topical serum provided comparable results versus a single treatment with a fractional CO_2_ laser system. Given the benefits demonstrated with the topical serum compared to the single fractional CO_2_ treatment, additional studies are warranted to further investigate the synergistic or complimentary benefits of the topical serum with such treatment, or with other laser treatments.

While no statistically significant differences in tolerability were observed, treatment with the topical cosmetic product achieved parity or statistically significant improvement in all measured parameters compared to laser treatment. By 16 weeks, both Laser and Topical Serum Cells had significant visual or tactile improvements for various parameters, including the expert grading parameters lines—global face and wrinkles—global face. Comparisons between the Laser Cell and the Topical Serum Cell showed that the Topical Serum Cell had comparable results as the Laser Cell after 16 weeks for various parameters. These data show that the tested topical serum is an effective way to combat visible signs of skin aging with results on multiple parameters that are comparable to a single laser treatment after 16 weeks of using the serum.

## Conflicts of Interest

The authors declare no conflicts of interest.

## Data Availability

The data that support the findings of this study are available on request from the corresponding author. The data are not publicly available due to privacy or ethical restrictions.

## References

[1] E. C. Naylor , R. E. B. Watson , and M. J. Sherratt , “Molecular Aspects of Skin Ageing,” Maturitas 69, no. 3 (2011): 249–256. 21612880 10.1016/j.maturitas.2011.04.011

[2] M. Ramos‐e‐Silva , D. M. Hexsel , M. S. Rutowitsch , and M. Zechmeister , “Hydroxy Acids and Retinoids in Cosmetics,” Clinics in Dermatology 19, no. 4 (2001): 460–466. 11535389 10.1016/s0738-081x(01)00189-4

[3] A. M. Darland , H. A. Chubb , D. L. Sachs , and Y. R. Helfrich , “Patient Interest in and Familiarity With Anti‐Aging Therapies: A Survey of the General Dermatology Clinic Population,” Journal of Cosmetic Dermatology 17, no. 3 (2018): 403–409. 28776925 10.1111/jocd.12386

[4] M. A. Farage , K. W. Miller , P. Elsner , and H. I. Maibach , “Functional and Physiological Characteristics of the Aging Skin,” Aging Clinical and Experimental Research 20, no. 3 (2008): 195–200. 18594185 10.1007/BF03324769

[5] M. Alexiades‐Armenakas , D. Sarnoff , R. Gotkin , and N. Sadick , “Multi‐Center Clinical Study and Review of Fractional Ablative CO_2_ Laser Resurfacing for the Treatment of Rhytides, Photoaging, Scars and Striae,” Journal of Drugs in Dermatology 10, no. 4 (2011): 352–362. 21455544

[6] P. K. Farris , B. L. Edison , R. L. Weinkauf , and B. A. Green , “A Novel, Volumizing Cosmetic Formulation Significantly Improves the Appearance of Target Glabellar Lines, Nasolabial Folds, and Crow's Feet in a Double‐Blind, Vehicle‐Controlled Clinical Trial,” Journal of Drugs in Dermatology 13, no. 1 (2014): 41–46. 24385118

[7] C. E. M. Griffiths , T. S. Wang , T. A. Hamilton , J. J. Voorhees , and C. N. Ellis , “A Photonumeric Scale for the Assessment of Cutaneous Photodamage,” Archives of Dermatology 128, no. 3 (1992): 347–351. 1550366

[8] J. Tan , Y. Lei , H. W. Ouyang , and M. H. Gold , “The Use of the Fractional CO_2_ Laser Resurfacing in the Treatment of Photoaging in Asians: Five Years Long‐Term Results,” Lasers in Surgery and Medicine 46, no. 10 (2014): 750–756. 25400224 10.1002/lsm.22304

[9] V. Comeau , M. Goodman , M. M. Kober , and C. Buckley , “Fractionated Carbon Dioxide Laser Resurfacing as an Ideal Treatment Option for Severe Rhinophyma: A Case Report and Discussion,” Journal of Clinical and Aesthetic Dermatology 12, no. 1 (2019): 24–27. PMC6405248 30881573

[10] K. L. Serowka , N. Saedi , J. S. Dover , and C. B. Zachary , “Fractionated Ablative Carbon Dioxide Laser for the Treatment of Rhinophyma,” Lasers in Surgery and Medicine 46, no. 1 (2014): 8–12. 24123064 10.1002/lsm.22184

[11] A. A. Meesters , M. M. D. van der Linden , M. A. de Rie , and A. Wolkerstorfer , “Fractionated Carbon Dioxide Laser Therapy as Treatment of Mild Rhinophyma: Report of Three Cases,” Dermatologic Therapy 28, no. 3 (2015): 147–150. 25753618 10.1111/dth.12205

[12] F. Urdiales‐Gálvez , S. Martín‐Sánchez , M. Maíz‐Jiménez , A. Castellano‐Miralla , and L. Lionetti‐Leone , “Concomitant Use of Hyaluronic Acid and Laser in Facial Rejuvenation,” Aesthetic Plastic Surgery 43, no. 4 (2019): 1061–1070. 31073742 10.1007/s00266-019-01393-7 PMC6742610

[13] S. Shah and M. Alam , “Laser Resurfacing Pearls,” Seminars in Plastic Surgery 26, no. 3 (2012): 131–136. 23904821 10.1055/s-0032-1329417 PMC3580978

[14] M. S. Ferreira , M. C. Magalhães , J. M. Sousa‐Lobo , and I. F. Almeida , “Trending Anti‐Aging Peptides,” Cosmetics 7, no. 4 (2020): 91.

[15] S. Mukherjee , A. Date , V. Patravale , H. C. Korting , A. Roeder , and G. Weindl , “Retinoids in the Treatment of Skin Aging: An Overview of Clinical Efficacy and Safety,” Clinical Interventions in Aging 1, no. 4 (2006): 327–348. 18046911 10.2147/ciia.2006.1.4.327 PMC2699641

[16] Ł. Szymański , R. Skopek , M. Palusińska , et al., “Retinoic Acid and Its Derivatives in Skin,” Cells 9, no. 12 (2020): 2660. 33322246 10.3390/cells9122660 PMC7764495

[17] C. E. Griffiths , L. J. Finkel , M. G. Tranfaglia , T. A. Hamilton , and J. J. Voorhees , “An In Vivo Experimental Model for Effects of Topical Retinoic Acid in Human Skin,” British Journal of Dermatology 129, no. 4 (1993): 389–394. 8217750 10.1111/j.1365-2133.1993.tb03163.x

[18] Ž. Temova Rakuša , P. Škufca , A. Kristl , and R. Roškar , “Retinoid Stability and Degradation Kinetics in Commercial Cosmetic Products,” Journal of Cosmetic Dermatology 20, no. 7 (2021): 2350–2358. 33206444 10.1111/jocd.13852

[19] Ž. Temova Rakuša , P. Škufca , A. Kristl , and R. Roškar , “Quality Control of Retinoids in Commercial Cosmetic Products,” Journal of Cosmetic Dermatology 20, no. 4 (2021): 1166–1175. 32813932 10.1111/jocd.13686

[20] J. C. Hubinger , “Determination of Retinol, Retinyl Palmitate, and Retinoic Acid in Consumer Cosmetic Products,” Journal of Cosmetic Science 60, no. 5 (2009): 485–500. 19822106

[21] N. Ruth and T. Mammone , “1310 Anti‐Aging Effects of Retinoid Hydroxypinacolone Retinoate on Skin Models,” Journal of Investigative Dermatology 138, no. 5, Supplement (2018): S223.

[22] D. Bai , F. Hu , H. Xu , et al., “High Stability and Low Irritation of Retinol Propionate and Hydroxypinacolone Retinoate Supramolecular Nanoparticles With Effective Anti‐Wrinkle Efficacy,” Pharmaceutics 15, no. 3 (2023): 1–14. 10.3390/pharmaceutics15030731 PMC10051651 36986592

[23] T. S. Alster and R. J. Hirsch , “Single‐Pass CO_2_ Laser Skin Resurfacing of Light and Dark Skin: Extended Experience With 52 Patients,” Journal of Cosmetic and Laser Therapy 5, no. 1 (2003): 39–42. 12745598

[24] G. Alvarez , N. Nath , and A. Suggs , “Real World Dermatology: Evaluating the Safety of Combination Laser Procedures in a Single Clinic Session,” Journal of Drugs in Dermatology 21, no. 11 (2022): 1181–1184. 36342729 10.36849/JDD.6766

[25] J. M. Knight and G. Kautz , “Sequential Facial Skin Rejuvenation With Intense Pulsed Light and Non‐Ablative Fractionated Laser Resurfacing in Fitzpatrick Skin Type II–IV Patients: A Prospective Multicenter Analysis,” Lasers in Surgery and Medicine 51, no. 2 (2019): 141–149. 30091207 10.1002/lsm.23007 PMC6585794

